# Clinical and inflammatory biomarkers predicting emergency surgical management decisions and postoperative outcomes in complicated colon cancer

**DOI:** 10.25122/jml-2026-0008

**Published:** 2026-04

**Authors:** Adrian-Marius Silaghi, Dragos Serban, Horia Pantu, Ion Motofei, Irina Shevchenko, Dimitrie-Ionut Atasiei, Simona Raluca Iacoban, Silviu Cristian Voinea, Catalin Cicerone Grigorescu, Vlad-Denis Constantin

**Affiliations:** 1 Doctoral School, Carol Davila University of Medicine and Pharmacy, Bucharest, Romania; 2 Department of General Surgery, St. Pantelimon Emergency Clinical Hospital, Bucharest, Romania; 3 Faculty of Medicine, Carol Davila University of Medicine and Pharmacy Bucharest, Bucharest, Romania; 4 Fourth General Surgery Department, Emergency University Hospital Bucharest, Bucharest, Romania; 5 Neurosurgery Department, Sf. Pantelimon Emergency Clinical Hospital, Bucharest, Romania; 6 Department of Obstetrics and Gynecology, Suceava County Hospital, Suceava, Romania; 7 Department of Oncological Surgery, Carol Davila University of Medicine and Pharmacy, Bucharest, Romania; 8 Department of Oncological Surgery, Alexandru Trestioreanu Oncology Institute, Bucharest, Romania; 9 Faculty of Medicine, Vasile Goldis Western University of Arad, Arad, Romania

**Keywords:** emergency surgery, neutrophil-to-lymphocyte ratio, stoma formation, postoperative complications, anastomotic leak, complicated colon cancer, bowel perforation, systemic immune-inflammation index, ASA, American Society of Anesthesiologists, AUC, area under the curve, BMI, body mass index, CCI, Charlson Comorbidity Index, CI, confidence interval, CRP, C-reactive protein, CT, computed tomography, GI, gastrointestinal, ICU, intensive care unit, IL, interleukin, NLR, neutrophil-to-lymphocyte ratio, OR, odds ratio, ROC, receiver operating characteristic, ROS, reactive oxygen species, SD, standard deviation, SII, systemic immune-inflammation index, SIRS, systemic inflammatory response syndrome, TNF-α, tumor necrosis factor alpha, VEGF, vascular endothelial growth factor

## Abstract

Emergency surgery for complicated colon cancer is associated with high morbidity and mortality, and selecting between primary anastomosis and stoma formation remains challenging. Systemic inflammation may influence intraoperative decisions and postoperative outcomes, but its role in emergency settings is not fully established. This retrospective study included patients undergoing emergency surgery for complicated colon cancer between October 2020 and January 2025. Patients were grouped according to surgical strategy (primary anastomosis vs. stoma formation). Preoperative inflammatory biomarkers—C-reactive protein (CRP), neutrophil-to-lymphocyte ratio (NLR), and systemic immune-inflammation index (SII)—were analyzed in relation to surgical decisions, postoperative complications, anastomotic leakage, and 30-day mortality using multivariate logistic regression and ROC curve analyses. Among 221 patients, 136 (61.5%) underwent primary anastomosis and 85 (38.5%) stoma formation. Higher CRP, NLR, and blood glucose levels were independently associated with a lower likelihood of primary anastomosis (AUC = 0.764). Postoperative complications occurred in 40.7% of patients and were independently predicted by body mass index, Charlson Comorbidity Index, serum creatinine, blood glucose, and NLR (AUC = 0.851). Anastomotic leakage was strongly associated with elevated CRP, NLR, SII, and serum creatinine, with CRP showing the highest predictive accuracy (AUC = 0.894). Elevated NLR and serum creatinine independently predicted 30-day mortality. Preoperative inflammatory biomarkers, particularly CRP and NLR, offer important prognostic value for surgical decision-making and early postoperative outcomes in emergency colon cancer surgery.

## Introduction

Complicated colon cancer represents a common indication for emergency laparotomy, accounting for approximately 5–10% of cases [1]. Surgical procedures in these situations represent the primary management option; however, they are associated with a high risk of postoperative complications such as anastomotic leaks [2], pneumonia [3], delayed wound healing [4], and increased 30-day mortality rate [5]. Under such conditions, stoma formation may represent the safer surgical option [6]. Still, in selected cases, primary anastomosis may be considered, especially in right-sided colon cancer, even in the presence of perforation [7]. Conversely, if the left-sided colon is affected, the decision to perform a primary anastomosis remains more controversial and should be individualized based on the severity of the systemic inflammatory response and the patient’s physiological reserve [8].

Compared with non-neoplastic causes of intestinal perforation or obstruction, oncologic patients exhibit a distinct and more pronounced inflammatory profile characterized by increased production of tumor necrosis factor alpha (TNF-α), interleukin-6 (IL-6), interleukin-8 (IL-8), interleukin-10 (IL-10), and vascular endothelial growth factor (VEGF) [9] due to presence of malignant cells and high bacterial burden [10]. For example, patients with colon cancer who present with bowel obstruction or local perforation have 1.7- and 2.2-fold higher concentrations of interleukin-1 (IL-1) and IL-6, respectively, compared with patients without complications [11]. The presence of proinflammatory cytokines such as IL-1, IL-6, and TNF-α, as well as reactive oxygen species (ROS), leads to a series of events [12], including the proliferation of granulocyte precursors in the bone marrow, the massive release into the systemic circulation, and the subsequent infiltration of the tumor microenvironment [13].

The activation of the NLRP1 inflammasome is another consequence of the massive release of inflammatory cytokines and can trigger caspase activation, which is associated with a decrease in circulating lymphocyte count, thereby altering the cellular immune response and increasing susceptibility to infections and delayed healing [14].

In clinical practice, these inflammatory events are reflected by elevated C-reactive protein (CRP) levels, accompanied by increased circulating neutrophils, macrophages, and monocytes. This inflammatory response promotes an excessive release of reactive oxygen species and is often associated with lymphopenia, leading to alterations in the neutrophil-to-lymphocyte ratio (NLR) and systemic immune-inflammation index (SII). Although these biomarkers have been used in elective surgery to predict long-term outcomes, it remains unclear whether they can reliably predict early postoperative complications in the emergency setting or assist surgeons in deciding between primary anastomosis and stoma formation, particularly regarding the risk of anastomotic leakage.

The present study aimed to explore the potential role of preoperative clinical and laboratory-derived inflammatory biomarkers, including SII and NLR, to guide surgical decision-making regarding the feasibility of primary anastomosis in patients undergoing emergency surgery for colon cancer. Secondarily, the study investigated potential associations between these biomarkers and early postoperative complications and 30-day mortality.

## Material and Methods

A retrospective comparative study was conducted in the Department of General Surgery at Saint Pantelimon Emergency Clinical Hospital, Bucharest, Romania. Patients who underwent emergency surgery for colon cancer between October 2020 and January 2025 were included and stratified into two groups according to the surgical approach: primary anastomosis and stoma formation.

Demographic and clinical variables (including body mass index [BMI] and type of presentation), laboratory and imaging data (such as complete blood count, computed tomography, and abdominal ultrasound), surgical details (type of procedure, tumor location, presence of perforation, and metastatic disease), and postoperative outcomes were collected from medical records and hospital database. The study included adult patients aged over 18 years who presented in an emergency setting with bowel obstruction, colon perforation, or gastrointestinal bleeding secondary to a primary colon tumor, underwent surgical resection, and in whom the diagnosis was confirmed by histopathological examination of the malignant tissue.

Patients included in the study underwent serological evaluation, which included complete blood count, assessment of hepatic and renal function, coagulation profile, blood glucose levels, and C-reactive protein. Subsequently, the inflammatory indices NLR and SII were calculated.

A multidisciplinary evaluation was conducted by the general surgeon and included an anesthesiologist and a cardiologist, with assessment of the Charlson Comorbidity Index (CCI) and the American Society of Anesthesiologists (ASA) score. In cases where patients met the criteria for sepsis or septic shock, management was conducted in accordance with the Surviving Sepsis Campaign guidelines [15]. Subsequently, patients were transferred to the operating theater and underwent surgery within the first 4 hours of hospital admission. Depending on the presence of sepsis criteria, comorbidities, and intraoperative findings, surgical procedures ranged from segmental colectomies to subtotal or total colectomies. Primary anastomoses were performed as end-to-end or side-to-side reconstructions, using absorbable monofilament sutures, and stoma formation was performed in accordance with established surgical standards and internationally accepted surgical guidelines.

Following surgery, patients were transferred to the Intensive Care Unit (ICU), where they were closely monitored for the development of postoperative complications. The presence of anastomotic leaks, wound dehiscence, and stoma-related complications was registered in the hospital information system and classified according to the Clavien–Dindo scale.

Exclusion criteria were represented by cases of uncomplicated colon cancer, local tumor recurrence, metastatic involvement of the colon originating from other primary malignancies, death before completion of surgery, internal bypass procedures without tumor resection, pregnancy, and age under 18 years.

Statistical analyses were performed using IBM SPSS Statistics version 26. Continuous variables were expressed as means ± standard deviations (SD) and compared using the Student’s *t*-test or ANOVA, as appropriate. Categorical variables were reported as frequencies and percentages and compared using the Chi-square (χ^2^) test or Fisher’s exact test. Odds ratios (ORs) with 95% confidence intervals (CIs) were also calculated. To identify independent factors influencing surgical decision-making, a multivariable binary logistic regression analysis was conducted, including variables deemed clinically relevant or associated with anastomosis in the univariate analysis (*P* < 0.10). Receiver operating characteristic (ROC) curve analyses were used to assess the ability of CRP, NLR, and SII to predict early postoperative complications and 30-day mortality. Optimal cutoff values were determined using the Youden index, and a two-tailed *P* value <0.05 was considered statistically significant.

## Results

After applying the inclusion and exclusion criteria, a total of 221 patients were included in the study, comprising 93 women and 128 men, aged between 37 and 94 years, with a mean age of 69.57 years. The mean BMI of the study population was 28.2, with values ranging from 15.33 to 43.25. Charlson Comorbidity scores ranged from 2 to 14, with a mean value of 6.76.

Of the 221 patients included in the study, 136 (61.5%) underwent primary anastomosis, while 85 patients (38.5%) required a stoma. The two groups showed statistically significant differences in terms of age, BMI, and CCI (*P* < 0.05). Patients who required stoma formation more frequently presented with systemic inflammatory response syndrome (SIRS), single or multiple organ failure, and required vasopressor support in comparison with those with primary anastomosis. A detailed comparison between groups is provided in Table 1.

**Table 1:** General characteristics of the patients

Characteristics	Primary anastomosis (*n* = 136)	Stoma formation (*n* = 85)	*P* value
Gender			0.203
Female	57 (41.9%)	36 (43.3%)	
Male	79 (58%)	49 (57.6%)	
Admission diagnosis			0.001
Bowel obstruction	72 (52.9%)	49 (57.6%)	
Peritonitis	18 (13.2%)	26 (30.6%)	
GI bleeding	46 (33.8%)	10 (11.8%)	
Age (years)	68.3 ± 11.1	71.6 ± 12.2	0.043
BMI (kg/m^2^)	27.7 ± 4.3	29.0 ± 5.5	0.049
CCI	6.5 ± 2.0	7.2 ± 2.0	0.012
CRP (mg/L)	62.1 ± 66.2	139.6 ± 123.9	< 0.001
NLR	6.2 ± 4.9	10.4 ± 7.0	< 0.001
SII	1514.5 (922.7- 2780.2)	2530.5 (1597.5–5151.5)	<0.001
Blood glucose (mg/dL)	126.1 ± 40.4	160.2 ± 71.1	< 0.001
Creatinine (mg/dL)	1.11 ± 0.72	1.45 ± 0.75	< 0.001
Vasopressor use			< 0.001
No	121(89.0%)	44(51.8%)	
Yes	15(11.0%)	41(48.2%)	
SIRS			< 0.001
No	115 (84.6%)	45 (52.9%)	
Yes	21(15.4%)	40(47.1%)	
Organ failure			<0.001
No	112(82.4%)	40(47.1%)	
Yes	24(17.6%)	45(52.9%)	
ASA			0.003
ASA ≤ 3	84 (61.8%)	36 (42.4%)	
ASA > 3	52 (38.2%)	49 (57.6%)	

On multivariate logistic regression analysis, higher preoperative NLR (OR = 1.079, 95% CI, 1.004–1.159; *P* = 0.038), CRP (OR = 1.006, 95% CI, 1.002–1.010; *P* = 0.005), and blood glucose levels (OR = 1.008, 95% CI, 1.002–1.015; *P* = 0.008) were associated with a lower likelihood of performing primary anastomosis. In contrast, BMI, Charlson Comorbidity Index, serum creatinine, and SII did not retain independent significance in the adjusted model. Also, the multivariate model demonstrated good discriminatory performance for primary anastomosis (AUC = 0.764)

The type of surgical resection and tumor location influenced the decision of primary anastomosis. Left-sided resections and left-sided colon tumors were more frequently accompanied by stoma formation compared with right-sided resections and tumors (χ^2^ = 60.15, *P* < 0.001; OR = 12.83, 95% CI, 6.40–25.71). In contrast, T and N stages did not significantly influence the decision to perform primary anastomosis (*P* > 0.05). A complete characterization of the intraoperative aspects is presented in Table 2.

**Table 2:** Intraoperative characteristics and surgical strategy of the patients

Patients’ characteristics	Primary anastomosis (*n* = 136)	Stoma formation (*n* = 85)	*P value*
Localization			< 0.001
Right colon	95 (69.9%)	13 (15.3%)	
Left colon	41 (30.1%)	72 (84.7%)	
Type of procedure			< 0.001
Right hemicolectomy	60 (44.1%)	4 (4.7%)	
Extended right hemicolectomy	33 (24.3%)	2 (2.4%)	
Left hemicolectomy	28 (20.6%)	46 (54.1%)	
Subtotal/Total colectomy	15 (11.0%)	33 (38.8%)	
T stage			> 0.05
T3	63 (46.3%)	37 (43.5%)	
T4	73 (53.7%)	48 (56.5%)	
N stage			> 0.05
N0-N1	90 (66.2%)	54 (63.5%)	
N2-N3	46 (33.8%)	31 (36.5%)	

In our study, 90 (40.7%) patients developed surgical complications. The most frequent complications were surgical wound complications, 67 patients, 31 (22.7%) experienced anastomotic leaks, and 22 (25.8%) developed stoma-related complications. Overall, postoperative surgical complications were significantly more frequent among patients requiring stoma formation compared with those undergoing primary anastomosis (*P* < 0.001).

Patients with and without postoperative complications were comparable with respect to age and sex distribution (69.08 vs. 70.28 years; *P* = 0.454). In contrast, patients who developed postoperative complications exhibited significantly higher Charlson Comorbidity Index scores (7.58 vs. 6.20; *P* < 0.001) and increased body mass index (BMI = 30.13 vs. 26.88; *P* < 0.001). Furthermore, a higher proportion of patients in the complicated group had an ASA score greater than 3, a higher prevalence of systemic inflammatory response syndrome, a greater need for vasopressor support, and higher rates of organ failure, with all associations reaching statistical significance (all *P* < 0.001).

In the cohort of patients who developed postoperative complications, preoperative inflammatory and metabolic markers were significantly higher, including CRP (147.7 vs. 53.5 mg/L), NLR (10.3 vs. 6.1), SII (4754.8 vs. 2208.2), blood glucose (161.2 vs. 124.1 mg/dL), and serum creatinine (1.56 vs. 1.02 mg/dL), compared with patients without complications (all *P* < 0.001).

**Figure 1:**
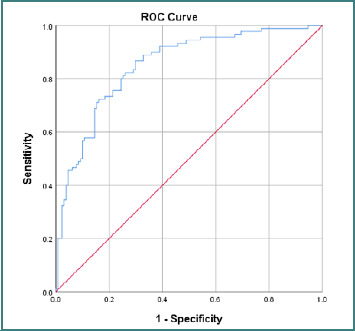
ROC curve of the multivariate model predicting postoperative complications

On multivariate logistic regression analysis, increased body mass index (OR = 1.16, 95% CI, 1.08–1.25; *P* < 0.001), higher Charlson Comorbidity Index (OR = 1.38, 95% CI, 1.15–1.65; *P* < 0.001), elevated serum creatinine (OR = 2.54, 95% CI, 1.51–4.28; *P* < 0.001), higher blood glucose levels (OR = 1.007, 95% CI, 1.001–1.014; *P* = 0.032), and increased NLR (OR = 1.06, 95% CI, 1.00–1.12; *P* = 0.037) were independently associated with the occurrence of postoperative surgical complications. The curve analysis of the multivariate model demonstrated excellent discriminative ability for predicting postoperative surgical complications, with an AUC of 0.851 (95% CI, 0.800–0.902; *P* < 0.001; Figure 1).

Regarding the patients who developed anastomotic leaks, preoperative inflammatory and clinical parameters were significantly higher, including CRP (150.2 ± 95.7 vs. 43.2 ± 37.4 mg/L), NLR (10.7 ± 5.8 vs. 5.3 ± 4.1), SII (6465.5 ± 6170.9 vs. 1744.2 ± 1580.7), blood glucose (153.7 ± 53.9 vs. 120.2 ± 34.4 mg/dL), and serum creatinine (1.95 ± 1.19 vs. 0.93 ± 0.38 mg/dL). BMI (29.5 ± 5.1 vs. 27.3 ± 4.0), Charlson Comorbidity Index (8.0 ± 1.5 vs. 6.2 ± 1.9), and ASA score (3.79 ± 0.42 vs. 3.16 ± 0.64) were also higher in comparison with those without anastomotic leaks (all *P* < 0.001).

**Figure 2:**
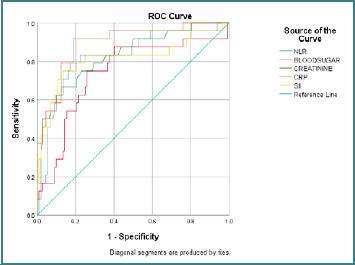
Receiver operating characteristic (ROC) curves of NLR, blood sugar, creatinine, CRP, and SII for predicting anastomotic leakage

ROC curve analysis performed in the anastomosis subgroup demonstrated excellent discriminatory ability for predicting anastomotic leakage (Figure 2). CRP showed the highest accuracy (AUC = 0.894), followed by serum creatinine (AUC = 0.832), NLR (AUC = 0.828), and SII (AUC = 0.814), while blood glucose showed moderate discrimination (AUC = 0.740) (all *P* < 0.001). Optimal cut-off values identified were CRP ≥ 64.36, NLR ≥ 7.12, creatinine ≥ 1.215, blood glucose ≥ 129.05, and SII ≥ 2895.64.

The overall mortality rate in our cohort was 25.33%, with deaths mainly related to septic complications (19.26%) and, to a lesser extent, to acute cardiac events (6.33%). In multivariate logistic regression analysis, preoperative NLR (OR = 1.10, 95% CI, 1.04–1.16; *P* = 0.001) and serum creatinine (OR = 2.63, 95% CI, 1.60–4.33; *P* < 0.001) emerged as independent predictors of mortality, whereas BMI, Charlson Comorbidity Index, and blood glucose did not retain statistical significance.

## Discussion

Emergency surgery is characterized by highly variable patient conditions and is associated with an increased risk of mortality and morbidity [16]. This risk is driven by the surgical aggression itself, which triggers an inflammatory response, and by the underlying pathology, which further exacerbates local and systemic inflammation [17]. Consequently, surgical decisions should be carefully considered and adequately justified. The primary intervention is ideally definitive, since any subsequent reoperation would occur in the context of persistent systemic inflammatory and metabolic disturbances [18].

The present study examined variations in inflammatory indices in patients presenting with bowel obstruction, perforation, and lower gastrointestinal bleeding secondary to colon tumors, and their influence on intraoperative management and postoperative outcomes. The accurate interpretation of these biomarkers depends on both rigorous statistical evaluation and an in-depth understanding of the pathophysiological processes underlying postoperative morbidity and mortality, thereby facilitating individualized management based on patient-specific factors.

The presence of tumor cells leads to a marked release of pro-inflammatory cytokines, IL-1, IL-6, IL-8, IL-17, and TNF-α, as well as chemokines such as CCL11 and CCL2. These mediators promote a local increase in neutrophils, macrophages, dendritic cells, and mast cells, as well as T and B lymphocytes. As a result, inflammatory cells may represent >50% of the cellular mass of the tumor [19]. Neutrophil infiltration of the extracellular matrix is associated with matrix metalloproteinase-9–mediated remodeling, promoting angiogenesis and inflammatory responses and potentially contributing to a pro-oncogenic environment [20]. The pro-inflammatory microenvironment, high tumor cell proliferation, and marked lymphocytic infiltration of tumor tissue may lead to a reduction in absolute lymphocyte count, which has been associated with an unfavorable long-term prognosis in patients with colon cancer [21]. Accordingly, the neutrophil-to-lymphocyte ratio has emerged as a robust biomarker for long-term prognostic stratification in colon cancer. High values are associated with decreased overall progression-free survival, poorer chemotherapy response, and higher rates of local recurrence [22]. Cutoff values for the neutrophil-to-lymphocyte ratio have been reported to range between 3 and 5. Higher NLR values have been reported in association with advanced-stage or metastatic disease [23,24].

In our cohort, preoperative neutrophil-to-lymphocyte ratio emerged as a consistent and clinically relevant biomarker across multiple decision-making and outcome domains. Higher NLR values were independently associated with a lower likelihood of primary anastomosis during emergency surgery (OR = 1.079, 95% CI, 1.004–1.159; *P* = 0.038). Accordingly, for each one-unit increase in preoperative NLR, the odds of performing primary anastomosis decreased by approximately 7.9%. In addition, elevated preoperative NLR remained an independent predictor of postoperative surgical complications (OR = 1.06, 95% CI, 1.00–1.12; *P* = 0.037) and demonstrated significant discriminatory performance for patients who developed anastomotic leakage. Importantly, higher NLR values were also independently associated with increased 30-day mortality (OR = 1.10, 95% CI, 1.04–1.16; *P* = 0.001).

When compared with previously reported cohorts that included patients with perforated colon cancer, our findings appear consistent. An elevated neutrophil-to-lymphocyte ratio (> 8.6) was associated with an increased incidence of postoperative complications, including ischemic events and anastomotic fistula, as well as higher 30-day mortality [25,26]. In contrast to patients undergoing emergency surgery for benign conditions, lower NLR values were observed; however, these values remained associated with short-term postoperative outcomes [27]. Collectively, these findings suggest that preoperative NLR integrates tumor-related inflammation with acute complications of colon cancer. Moreover, this inflammatory marker can influence the decision to perform a primary anastomosis and predict early postoperative outcomes (including anastomotic leaks) in emergency colon cancer surgery.

In addition to NLR, SII is another inflammatory index that incorporates multiple circulating cell line counts, such as neutrophil, lymphocyte, and platelet counts, and has been proposed as a comprehensive marker of systemic inflammatory burden in oncologic patients [28]. Thrombocytosis in colon cancer may result from increased interleukin-6 release, which stimulates hepatic synthesis of thrombopoietin and is associated with adverse outcomes [29]. Platelet–tumor cell interactions can activate inflammatory and pro-angiogenic pathways by releasing prostaglandin E_2_ and thromboxane A_2_, thereby promoting tumor cell survival, adhesion, invasion, and metastatic potential [30]. Furthermore, cancer-associated hypercoagulability may contribute to microthrombosis, impaired tissue healing, and altered immune responsiveness, resulting in high rates of anastomotic leaks, surgical wound infections, or deep vein thrombosis [31].

In our cohort, the systemic immune-inflammation index appeared to be more useful for risk stratification than as an independent predictor of surgical decision-making or early postoperative outcomes. In univariate analysis, higher SII values were associated with decreased possibility of primary anastomosis (OR = 1.012, 95% CI, 1.004–1.020; *P* = 0.004), higher risk of postoperative complications (OR = 1.022, 95% CI, 1.012–1.032; *P* < 0.001), and anastomotic leakage (OR = 1.051 95% CI, 1.024–1.077; *P* < 0.001). However, in multivariable logistic regression analysis assessing the likelihood of primary anastomosis versus stoma formation, SII did not retain independent predictive value (OR = 0.993, 95% CI, 0.981–1.005; *P* = 0.206). Similarly, when adjusted for NLR, the association between SII and postoperative complications was attenuated (OR = 1.005, 95% CI, 0.991–1.019; *P* = 0.488), suggesting overlapping prognostic information.

In contrast, SII demonstrated good discriminatory performance for anastomotic leakage on receiver operating characteristic curve analysis (AUC = 0.814, 95% CI, 0.695–0.932; *P* < 0.001). These results suggest that SII may assist surgeons in intraoperative decision-making regarding primary anastomosis versus stoma formation and in identifying patients at increased risk of postoperative complications, particularly anastomotic leakage. However, its prognostic effect is attenuated after adjustment for other inflammatory markers.

Alongside cellular inflammatory markers, C-reactive protein is a widely used parameter that reflects the magnitude and severity of the inflammatory response. C-reactive protein is an acute-phase reactant synthesized in the liver cells, and its release to the bloodstream is primarily influenced by interleukin-6 [32]. In sepsis trauma, or hypoxia, a marked increase in CRP levels occurs in the first 6 hours, and after 24–72 hours, the peak value is reached [33].

The association between elevated C-reactive protein levels and the progression of colon cancer is closely linked to the pro-tumoral microenvironment. Under conditions of oxidative stress and sustained exposure to pro-inflammatory mediators, including interleukin-6, epithelial–mesenchymal transition is enhanced, concomitant with increased hepatic synthesis of C-reactive protein, which will exert a positive feedback effect on endothelial cells and neutrophils, promoting epithelial–mesenchymal transition and the activation of nuclear factor-κB involved in the proliferation of the malignant cells [34]. Collectively, these mechanisms have been associated with reduced overall survival [35].

Among the inflammatory markers evaluated, C-reactive protein demonstrated the strongest and most consistent prognostic value, retaining independent significance in intraoperative decision-making, regarding a reduced likelihood of performing primary anastomosis (OR = 1.006, 95% CI, 1.002–1.010; *P* = 0.005). Moreover, CRP levels were markedly higher in patients who developed anastomotic leakage than in those who did not (150.2 ± 95.7 vs. 43.2 ± 37.4 mg/L) and showed excellent discriminative performance on receiver operating characteristic curve analysis (AUC = 0.894).

Our findings are consistent with previously published data. In their meta-analysis of elective colorectal surgery, McKechnie *et al*. demonstrated that elevated preoperative C-reactive protein levels were associated with an increased risk of infectious complications, including anastomotic leakage [36]. Despite these similarities, the strength of the association was attenuated, possibly due to differences in patient selection and clinical context between elective and emergency cohorts.

The decision to perform a primary anastomosis in patients with complicated colon cancer remains challenging. In the emergency setting, patients with complicated colon cancer are often elderly and burdened by a higher Charlson Comorbidity Index, which has proven predictive value for 5-year mortality [37], risk of developing anastomotic leaks [38], and other postoperative complications related to orthopedic [39] or gastrointestinal procedures [40]. In addition, many require vasopressor agents or meet the criteria for SIRS/septic shock [41]. All of these factors are well-recognized contributors to an increased risk of anastomotic leakage. In such circumstances, stoma formation is frequently preferred [42] as the occurrence of postoperative complications may have profound and potentially life-threatening consequences in patients with limited physiological reserve.

In our cohort, patients who underwent stoma formation had significantly higher Charlson Comorbidity Index scores than those receiving primary anastomosis (7.2 ± 2.0 vs. 6.5 ± 2.0; *P* = 0.012), as well as a higher proportion with ASA class > 3 (57.6% vs. 38.2%; *P* = 0.003). Moreover, systemic inflammatory and hemodynamic compromise was more frequently observed in the stoma group, with higher rates of systemic inflammatory response syndrome (47.1% vs. 15.4%; *P* < 0.001), organ failure (52.9% vs. 17.6%; *P* < 0.001), and vasopressor requirement (48.2% vs. 11.0%; *P* < 0.001) These findings are in accordance with other studies [43,44] and confirm the role of these clinical and physiological factors as major determinants in the decision not to perform primary anastomosis.

According to our analysis, inflammatory biomarkers should not be interpreted as isolated decision-making tools, but rather as parameters that complement clinical judgment and, in conjunction with intraoperative findings, may optimize individualized patient management. In this context, preoperative CRP appears to provide the most robust biological signal, while NLR and SII may offer complementary information for patient stratification, particularly in borderline cases where surgical decision-making is challenging.

This study has several limitations. First, its retrospective design and single-center nature may limit the generalizability of the findings to broader patient populations. Second, inflammatory markers were assessed at a single preoperative time point, and dynamic postoperative trends were not evaluated. In addition, accurate documentation of anastomotic leakage or other postoperative complications can be challenging. The occurrence of adverse postoperative events may have a significant psychological impact on surgeons, potentially leading to sentiments of guilt, stress, anxiety, and depressive symptoms. As a result, some complications may be underreported, either as a coping mechanism to mitigate personal psychological distress or due to concerns related to potential medico-legal consequences [45].

Future studies should evaluate these inflammatory parameters dynamically to improve their accuracy and integrate them with clinical and intraoperative variables to better guide decision-making regarding primary anastomosis.

## Conclusion

Emergency surgery for complicated colon cancer is associated with substantial morbidity and mortality, and intraoperative decision-making regarding primary anastomosis remains particularly challenging. The results of this study demonstrate that preoperative inflammatory biomarkers provide valuable information for both surgical strategy and risk stratification in this high-risk population. Elevated preoperative CRP, NLR, and SII levels may assist surgeons in decision-making regarding the feasibility of primary anastomosis, enable discrimination between patients with and without anastomotic leakage, and help identify those at increased risk of postoperative complications.
